# Effects of Occupational Therapy via Telerehabilitation on Occupational Balance, Well-Being, Intrinsic Motivation and Quality of Life in Syrian Refugee Children in COVID-19 Lockdown: A Randomized Controlled Trial

**DOI:** 10.3390/children9040485

**Published:** 2022-04-01

**Authors:** Sümeyye Belhan Çelik, Esma Özkan, Gonca Bumin

**Affiliations:** 1 Department of Occupational Therapy, Hamidiye Faculty of Health Sciences, University of Health Sciences Turkey, İstanbul 34668, Turkey; 2 Department of Occupational Therapy, Institute of Health Sciences, Hacettepe University, Ankara 06050, Turkey; gbumin@hacettepe.edu.tr; 3 Department of Occupational Therapy, Gülhane Faculty of Health Sciences, University of Health Sciences Turkey, Ankara 34668, Turkey; esma.ozkan@sbu.edu.tr

**Keywords:** occupational therapy, refugee, children, occupational balance, quality of life, well-being, COVID-19

## Abstract

We aimed to evaluate the effects of an occupational training program via telerehabilitation on well-being (WB), occupational balance (OB), intrinsic motivation (IM), and quality of life (QoL) in Syrian refugee children resettled in Turkey during the COVID-19 pandemic. This was a single-center, prospective, randomized, non-blinded trial in which children aged 13–15 years and attending a secondary school were recruited. OB, WB, IM, and QoL were evaluated via the OB Questionnaire (OBQ11), the Well-Star Scale (WSS), the IM Scale (IMS), and the Pediatric Quality of Life Inventory (PedsQL). The intervention group attended online occupational therapy classes. Online classes were carried out as five sessions per week, each session lasting 1 h, for 3 weeks. Questionnaires were performed at the outset of the study and following the training program. Overall, 52 refugee children were randomized into the intervention and control groups, each including 26 children. The mean OBQ11, WSS, IMS, and PedsQL scores significantly improved more in the intervention group than in the control group. This was the first study investigating the effects of a customized online training course on OB, WB, IM, and QoL in Syrian refugee children, also affected unfavorably by the COVID-19 lockdown. Our results showed significant improvements in all the study scales that we used to quantify the alterations in the aforementioned traits.

## 1. Introduction

The Syrian civil war has been raging for approximately 10 years. Unfortunately, at least 12,000 children have been killed or injured to date as a joint result of armed conflict, economic crisis, and the COVID-19 pandemic [1]. Due to the adversities in Syria, many families were forced to seek refuge from the neighboring countries, particularly Turkey. It is estimated that as of February 2021, Turkey was home to 1.6 million Syrian refugee children [2].

The advent of the first case of COVID-19 in Turkey in March 2020 made the situation more complicated, especially for refugees. Although the disease itself did not affect children as much as adults in terms of health hazards, it had a tremendous impact on school enrolment, attendance, and retention due to frequent lockdowns. Currently, it is estimated that 400,000 school-aged Syrian refugee children are out of school in Turkey [3]. For those who were fortunate enough to enroll in a school, the year 2020 was spent at home while formal education continued via state-wide broadcasting and online training by local attended schools.

Refugee children, especially in the example of Syria, experience a wide range of traumatic events before resettling in a host country including armed conflict and bombing, destruction of home, injury of self or beloved ones, witnessing death and violence, hunger, and protracted and dangerous journeys to safe places to mention a few [4,5]. It has been well established that children who experience war and related traumatic events have difficulties in cognitive skills such as attention, learning, retaining new information, memory, and executive functions. In addition, posttraumatic stress disorder (PTSD), anxiety, and depression are common occurrences among refugee children [6], which interfere with learning by direct and indirect mechanisms.

Several recent studies have shown that intervention programs might be effective to some extent in the amelioration of untoward effects of PTSD, anxiety, and depression on refugee children. Gormez and colleagues reported the efficiency of a cognitive-behavioral intervention on trauma-affected Syrian refugee children [7].

The COVID-19 pandemic made it harder for refugee children to cope with the difficulties with which they were already struggling. The lack of peer–teacher support when refugee children are in lockdown at home, the lack of motivation when trapped at home, and the imbalance in their activities affect the activity-role (occupational) balance, well-being, quality of life, and intrinsic motivation. As well as the war experiences, situations such as the adaptation process to remote education, interruption of face-to-face education, the disruption of the sleep–resting cycle, and balance in routine activities result in a decrease in occupational balance. Several studies have already demonstrated the adverse impact of social distancing during the COVID-19 pandemic on occupational balance [8]. In addition to the COVID-19 pandemic, displacement results in occupational dysfunction that puts occupational balance at risk [9]. Occupational balance is fundamental for well-being, happiness, motivation, quality of life, and health [10]. Occupational dysfunction is caused by occupational imbalance, which happens when everyday activities and tasks are not integrated in a pleasant manner. Individuals with poor occupational functionality struggle to participate in daily activities [11].

Well-being refers to the situation in which the material, cognitive, emotional, and spiritual power of the individual, family, or community is combined with positive socio-cultural relations and a positive economic and political environment [12]. While the physical, social, and psychological well-being of a large number of refugee children around the world has already been impacted by the negative effects of war and displacement, isolating children from their school life and/or social interactions with their peers during the COVID-19 pandemic isolation period may put their physical, social, and psychological well-being at risk [13].

Intrinsic motivation is one of the important determinants of well-being. Intrinsic motivation is defined as an individual partaking in an activity in order to naturally gain satisfaction. It is significant that the activity is interesting and enjoyable rather than driven by external rewards or pressures. In addition, the knowledge and abilities of the individual develop through their activities in parallel with their existing natural interests [14].

Quality of life—one of the most important concepts in mental health—encompasses a number of sub-concepts such as happiness, social well-being, and personal relationships. It is a broad concept that includes a person’s physical health, psychological state, level of independence, social relationships, personal beliefs, and their relationship with environmental characteristics [15]. The COVID-19 epidemic has altered the lives of billions of children and adolescents in dramatic and unprecedented ways. It is stated that even before the COVID-19 pandemic, 81% of students aged 11–17 were not physically active enough and inactivity was associated with an increased risk of obesity, activities based on the use of electronic devices, decreased physical condition, self-esteem, and prosocial behavior. Apart from inactivity, stressful events such as the COVID-19 pandemic have had a detrimental effect on children’s quality of life. Home quarantine circumstances, as well as the use of the internet or mobile phones, all contributed to the establishment of these conditions [16]. School closures, house arrest, and social distancing measures, especially during the pandemic period, may place a much greater burden on children and adolescents compared with other individuals. Childhood and adolescence are critical periods for social development with greater demands for social connection and the problems and consequences of COVID-19 can have a significant impact on their QoL and mental health. Therefore, it is critical to establish policies and programs such as educational intervention to improve learning in order to improve the quality of life of children, who are the most vulnerable population [17]. In this context, quality of life should be addressed in various areas that affect children’s lives such as education, social services, health services, and family policy. Quality of life should be a unifying term where the added value in addressing children’s rights and well-being includes the same domains for all children and a representation of their subjective experiences [18].

Occupational therapy, since its inception 100 years ago, has been demonstrated to benefit individuals with respect to health promotion, well-being, and quality of life [19]. This requirement is more imperative under the dual stresses of being a refugee and living in a COVID-19 lockdown. The COVID-19 pandemic also highlighted the importance of online deliverance of occupational therapy [20].

In summary, social distancing and lockdown associated with the COVID-19 pandemic might have complicated the already vulnerable situation of the refugee children. Cognitive and learning difficulties created by traumatic events related to difficulties and trauma experienced during the Syrian civil war and the resettlement period in Turkey might have been complicated by the limitations imposed by the COVID-19 pandemic. Thus, it is of the utmost importance to find out and implement effective and feasible ways of intervention to ameliorate adverse effects of the refugee experience and COVID-19 pandemic on cognitive functions, well-being, quality of life, and occupational balance in these children. To that purpose, we designed a randomized controlled trial in which we examined the effects of a customized occupational therapy training program via telerehabilitation encompassing various activities on the aforementioned aspects of refugee children resettled in Turkey.

Our hypotheses:

**H1.**  *Occupational therapy training* via *telerehabilitation in Syrian refugee children has no effect on occupational balance*.

**H2.**  *Occupational therapy training* via *telerehabilitation has no effect on well-being in Syrian refugee children*.

**H3.**  *Occupational therapy training* via *telerehabilitation has no effect on intrinsic motivation in Syrian refugee children*.

**H4.**  *Occupational therapy training* via *telerehabilitation has no effect on the quality of life in Syrian refugee children*.

## 2. Material and Methods

### 2.1. Design and Ethical Aspects

This was a single-center, prospective, randomized, non-blinded trial in which the effects of an online training course on occupational balance, well-being, intrinsic motivation and quality of life of refugee children were studied. The study was carried out at the secondary school. The study protocol was approved by the Hamidiye Non-invasive Investigation Ethics Committee (15 June 2020-20/213). Furthermore, this study was registered by the Registry of Clinical Trials (NCT05233345).

The study was performed in accordance with the Declaration of Helsinki and followed CONSORT guidelines [21]. The contact information of all potential study participants was obtained from the administration of the secondary school. Before enrolment, to inform the parents of the prospective participants, informed consent forms were prepared in which study aims and protocols were described in detail. Informed consent forms were signed by the study participants and their parents at a meeting with study investigators at their homes.

### 2.2. Participants and Eligibility Assessment

All refugee children, whose parents and themselves consented to participate in the study, aged between 13 and 15 years were included in the study. The recruitment process spanned between 13 December 2020 and 31 December 2020.

Inclusion criteria were as follows: aged between 13–15 years, having experienced war in home country, being a current resident in the Fatih province of Istanbul, being able to read and write (literate) in Turkish, living at home during the lockdown, attending online classes through the Ministry of National Education Online Education Platform (EBA), and the willingness of both the child and their family to participate in the study.

Exclusion criteria included: the presence of a known neurologic or developmental disorder or learning difficulty, or already participating in an additional education or activity program apart from the EBA.

**Figure 1:**
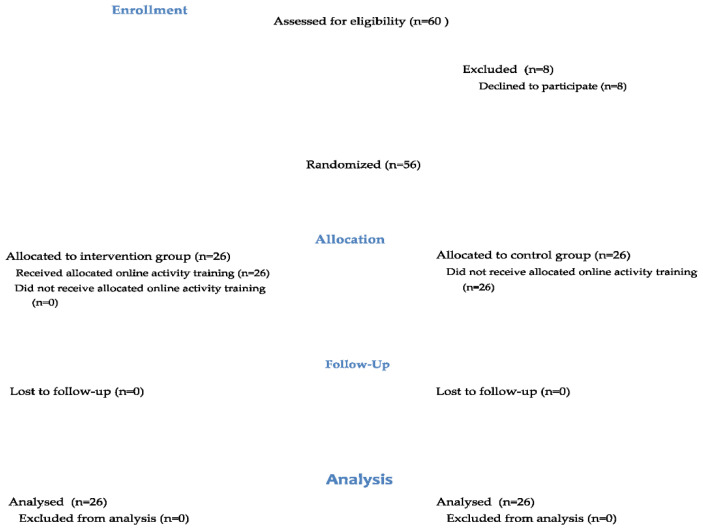
CONSORT flow diagram: the flow of children during each trial phase.

Overall, 60 refugee children were screened who met the age criteria at the secondary school. The demographic characteristics of the cases are given in Table 1 of the result section. Of these, 8 children were excluded due to not meeting inclusion criteria or unwillingness to be included in the study. Finally, 52 children (mean age 13.69 ± 0.87) were randomized into one of the two groups: group I (control group), EBA only; group II (intervention group), EBA + occupational therapy via telerehabilitation. Figure 1 is a flowchart of the children who participated during each trial phase: enrollment, allocation, follow-up, and data analysis.

### 2.3. Interventions

All study participants continued taking classes from the EBA program as part of their routine education plan. EBA is a public free online education platform created by the Ministry of National Education to enable students to access online education from their homes during the COVID-19 epidemic process, where education is carried out remotely. The group that received the occupational therapy classes via telerehabilitation, the effect of which was investigated, was the intervention group. Online classes were carried out as 5 sessions per week, each session lasting 1 h, for 3 weeks. In total, 15 sessions were performed during the study period.

Group activities (Table 2) were performed through the Zoom application by means of a video camera. Online group activities included painting and cake making together, sports activities to be performed simultaneously with the movements shown by the researcher, memory games, and games that can be played with the group such as the categories game.

At the same time, children were asked to interpret idioms and proverbs and to express what they understood from the book they needed to read daily by choosing a different person in the group every day. Sports activities comprised the movements demonstrated by the researcher through online Zoom meeting performed simultaneously with all group members. These sports activities were performed in the last 15 min of group activities 5 days a week in order to increase the physical activity level of children whose physical activities decreased during the lockdown period at home.

Apart from the online training to be held with the group, an occupational balance program chart (Table 3) was created for individuals and applied through a single-person online session to organize daily routines and occupations and to perform activities in a balanced way. This chart comprised the children’s activities hour by hour from waking up to bedtime. The children were asked to apply this chart 6 days a week; Sunday was considered a resting day. The routine daily chart included test solving (50 questions a day), reading a book, learning an idiom and a proverb, and playing 10 games from the MentalUp application. The MentalUp application is a free mind–intelligence game application that determines cognitive exercises that improve cognitive skills (attention, concentration, problem-solving skills, visual perception) at home according to the age of the user. In this application—that helps develop children’s thinking and learning skills and thus increases their academic performance—there are hundreds of games that improve children’s short-term memory, divided attention, sustained attention, reaction control, focus, mathematical skills, planning and organization skills, visual attention, visual scanning, and visual memory skills. If the child did not have his/her own phone, their parents downloaded this application to their phone. The child played 10 games from this application for 3 weeks, 6 days a week, and sent the score they got to the researcher via the WhatsApp application on a daily basis. The winner of the day was announced in the WhatsApp group—in which all the children were involved—to create a competitive environment and to ensure their motivation.

A score sheet was also prepared by the investigator. In order to ensure the continuity of the 3-week program in a motivated way by providing a competitive environment, one star was given to those who did the daily routine, and two stars were given to the child with the highest score in the MentalUp application. Likewise, starting at the same time in the group training, for example, the child who painted the most beautiful picture or made shapes from legumes and pasta and painted them in 15 min was chosen by voting by the group members and the winner of that online session was given two stars on the scoreboard. At the outset of the training, the volunteers were told that the stars would be counted at the end of the training and a prize would be given to those who received the most stars, thus increasing their integration into the program.

The daily activities in one sample week of the 3-week occupation-based training chart are shown in Table 3 and were continued in the same way for 3 weeks.

### 2.4. Measures

The primary outcome measure was the efficiency of the occupational therapy, which was assessed in terms of occupational balance. The secondary outcome measures were the quality of life, general well-being, and intrinsic motivation. To evaluate these traits, several questionnaires were used. All questionnaires were completed by the study participants at the start of the study (baseline evaluation) and repeated at the end of the 3-week study period. Questionnaires were applied online by one of the investigators, who was blinded to the group status of the participants.

The following questionnaires were used: the Occupational Balance Questionnaire, the Well-Star Scale, the Intrinsic Motivation Scale, and the Pediatric Quality of Life Inventory. Questions were asked by one of the investigators and answered by children through online video meetings.

#### 2.4.1. Occupational Balance Questionnaire (Revised Version, OBQ11)

This questionnaire is used to assess the activity–role balance according to the level of contention and to define the variety of the activities and occupational balance based on the results of the obtained data. A reliability and validity study of the Turkish version was conducted by Gunal et al., [22]. Each item in the OBQ is scored between 0 (strongly disagree) and 3 (strongly agree). The total score varies between 0 and 33 points. Higher scores indicate better occupational balance.

#### 2.4.2. The Well-Star Scale

The Well-Star Scale, developed by Korkut-Owen et al., [23], consists of 24 items and 5 dimensions. In its broadest meaning, well-being is a concept that means to be well in a number of dimensions of life and is explained by a series of models. This scale evaluates an individual’s well-being state, their ability to make sense of life, and how target oriented they are in the context of cognitive, emotional, physical, and social dimensions. The scores that can be obtained from the scale range between 24 and 120. Higher scores indicate that the individual views their well-being favorably. Sub scores of this scale can also be calculated in various dimensions.

#### 2.4.3. Intrinsic Motivation Scale (IMS)

The scale was developed primarily to assess individual differences in propensity toward intrinsic motivation in leisure behavior and was originally named as the “Intrinsic Motivation Scale” (ILM). A Turkish version reliability and validity study was conducted by Duman and colleagues [24]. The original version of the scale is composed of 24 items and 4 subscales. Seven numeric options are available to answer the scale questions between 1 (not at all true) and 7 (very true). For reverse scoring items 6, 13, and 18 item response was subtracted from 8 and then used as the result of that item. The maximum attainable score is 168 on the scale and the higher the total score the higher the level of intrinsic motivation.

### 2.5. Pediatric Quality of Life Inventory (PedsQL)

The Pediatric Quality of Life Inventory is used to assess the health-related quality of the life of the subjects. The reliability and validity study of the Turkish version was performed by Memik el al. [25]. Physical, emotional, social, and school functioning are among the key scales included in this survey. This inventory is a useful tool for assessing the health-related quality of life of healthy children and adolescents, as well as those with acute and chronic illnesses, in big groups such as schools and hospitals. The PedsQL inventory consists of 23 items that are scored between 0 and 100. When a question is answered as “never”, 100 points are given; “Almost never”, “sometimes”, “often”, and “almost always” answers are given 75, 50, 25, and 0 points, respectively. The items of the PedsQL cover school functioning and core dimensions of health described by WHO, including physical health, emotional functioning, and social functioning. Three summary scales are scored based on the answers given to inventory questions: total scale score (TSS), physical health summary score (PHSS), and psychosocial health summary score (PsHSS). Psychosocial health summary score is composed of scores obtained from the scales of emotional (EFS), social (SFS), and school functioning (ScFS) [26]. Higher PedsQL scores indicate the better health-related quality of life. Brevity, easy applicability, and scoring by the investigator and the short time needed to complete are important advantages of the inventory. Total PedsQL score = Arithmetic averages of PHSS and PHSS (PHSS = averages of EFS, SFS, and ScFS).

### 2.6. Randomization and Blinding

All study participants were randomly allocated to control or intervention groups by the type of simple randomization. Children who met the inclusion criteria were randomly assigned to the groups, with equal probability and independent of previous assignment, using the random number generator via computer software. At baseline and post testing, the researcher who made the pre- and post-training evaluations was blinded to the children’s group assignment. Pre- and post-assessments were made by the same researcher. Furthermore, as part of the treatment, online sessions were applied by another researcher therapist.

### 2.7. Statistical Analysis

Considering an 80% test power and α < 0.05, a minimum of 26 subjects was found to be needed for each group. Power analysis was conducted with the G* Power 3.1.9.2 software program based on the outcomes of Doumit et al. [27].

The Shapiro–Wilk test was used to check the normality of the continuous variables. Continuous variables were expressed as mean ± standard deviation or median (minimum: maximum) depending on the distribution of the variable. Based on the results of the normality test, the Mann–Whitney U test was used for intergroup comparisons. Analysis of categorical variables was performed with the Chi-squared test. We calculated a “difference score”, which was the difference between the scores of scales at the study outset and scores attained at the end of the study. These difference scores were compared by means of the Mann–Whitney U test and an independent samples *t*-test. To examine the change of difference scores in the intervention and control groups, a paired samples *t*-test and the Wilcoxon signed-rank test were used.

Statistical analyses were performed using SPSS (IBM Corp. Released 2012. IBM SPSS Statistics for Windows, Version 21.0. IBM Corp., Armonk, NY, USA). Type I error level was accepted as 5% in statistical analyses.

## 3. Results

Overall, 52 refugee children were included in the trial. After randomization, the intervention and control groups each included 26 children. The groups were comparable in terms of age, the number of siblings, and duration of resettlement in Turkey. We categorized the children in each group into subgroups based on the number of siblings and duration of living in Turkey; there was also not any significant difference between the intervention and control groups (Table 4).

### 3.1. Baseline Scores of the Study Scales

The mean scores of the Occupational Balance Questionnaire (OBQ11), the Well-Star Scale (WSS), the Intrinsic Motivation Scale (IMS), and the Pediatric Quality of Life Inventory were similar between the intervention and control groups at the outset of the study. The core scale scores of the PedsQL inventory were also not different between the groups. Table 5 depicts the baseline scores of the study scales before online training intervention.

### 3.2. Effect of Training via Telerehabilitation Intervention on Study Scales

**Figure 2:**
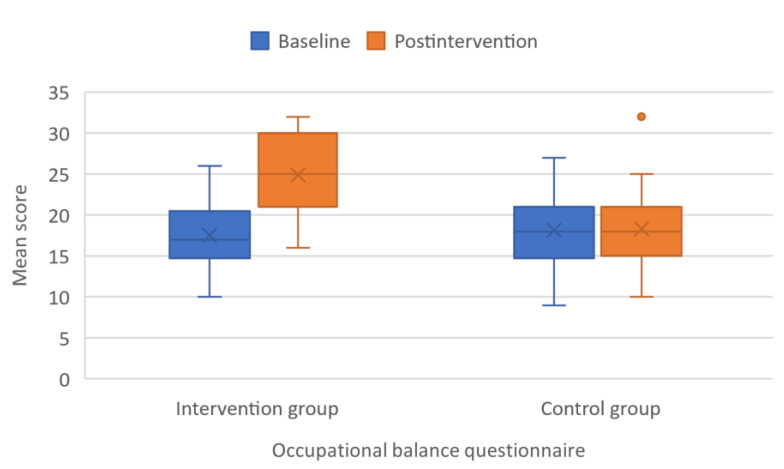
Boxplot with error bars showing mean scores of Occupational Balance Questionnaire (OBQ11) at baseline and after intervention.

Occupational balance improved significantly more in the intervention group compared with the control group. The mean OQB11 score increased 7.38 ± 3.31 and 0.19 ± 2.93 points in the intervention and control groups, respectively (*p* < 0.001) (Figure 2).

**Figure 3:**
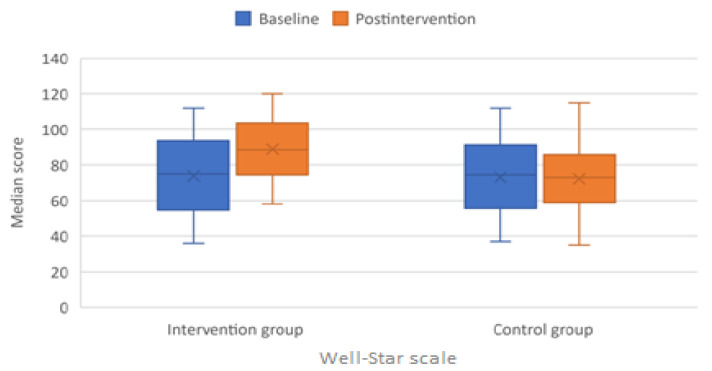
Boxplot graph depicting median scores of Well-Star scale (WSS) at baseline and after intervention.

The well-being of the children, which was assessed by the Well-Star scale, improved significantly in the intervention group compared with the control group (*p* < 0.001) (Figure 3). A median of 13.5 points increase in WSS was obtained in the intervention group versus only a median of 1 point in the control group.

**Figure 4:**
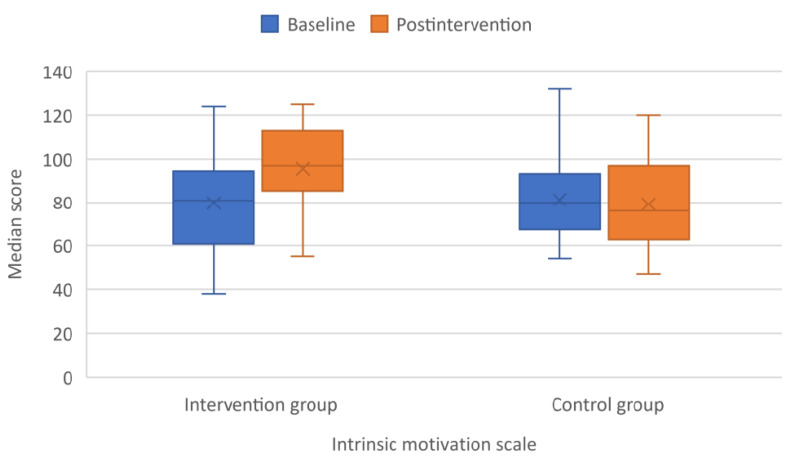
Boxplot graph depicting median scores of Intrinsic Motivation scale (IMS) at baseline and after intervention.

Children who underwent online training experienced a significant increase in their intrinsic motivation. The IMS score increased by a median of 17 points in the intervention group, whereas it decreased by a median of 2.5 points in the children who served as the control group (*p* < 0.001) (Figure 4).

**Figure 5:**
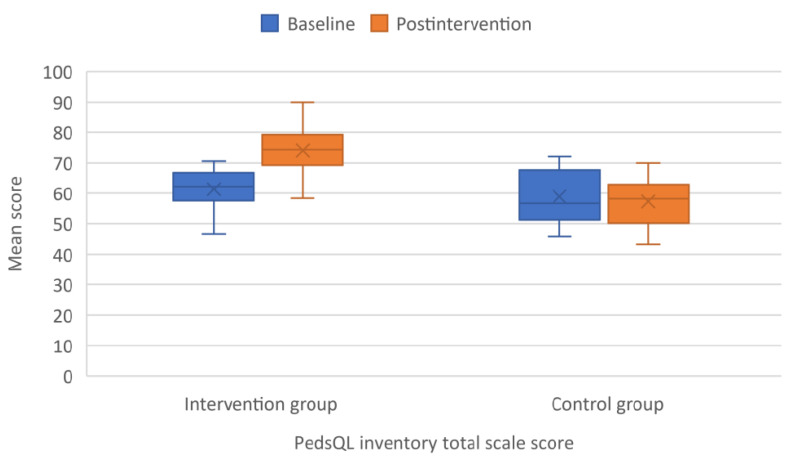
Boxplot with error bars showing mean scores of Pediatric Quality of Life Inventory (PedsQL) total scale score at baseline and after intervention.

The health-related quality of life of the children who underwent online training significantly improved compared with their counterparts who were included in the control group. The total scale score of the PedsQL inventory increased by a mean of 12.7 ± 5.7 points in the intervention group compared with a mean of −1.7 ± 5.2 in the control group (Figure 5). All core scales of the PedsQL in the intervention group showed a similar increase and improved significantly more than those of the children in the control group.

Table 6 summarizes baseline and post-intervention scores of study scales for the control and intervention groups. Comparison of “score differences” for each study scale between the control and the intervention groups are presented in Table 7.

## 4. Discussion

The salient findings of the present study can be summarized as follows: (a) online training intervention significantly improved the occupational balance of the refugee children, (b) well-being of the children was significantly improved by the occupational therapy via telerehabilitation, (c) the occupational therapy via telerehabilitation significantly increased the intrinsic motivation of the refugee children, finally (d) health-related quality of life of the participants who underwent the occupational therapy via telerehabilitation significantly improved compared with the control group. Overall, our results demonstrated the conspicuous benefits of the occupational therapy via telerehabilitation that was designed to augment the well-being, quality of life, and intrinsic motivation of refugee children.

When we compare our pre- and post-intervention data, we see that our telerehabilitation application has shown a considerable improvement in terms of the variables we measured. The COVID-19 pandemic rendered occupational therapy an online training method in many parts of the world; this change actually came as an emergency response to a crisis [28]. Several studies reported the utilization and effectiveness of telerehabilitation programs applied by occupational therapists [29]. Dahl-Popolizio et al., [30] evaluated how occupational therapy practitioners (OTP) handled telehealth during the COVID-19 pandemic. The results of the cross-sectional study exhibited that 77% of 230 OTPs supported telerehabilitation programs as an appropriate substitute to face-to-face training. The results of our study show that occupational therapy practices via telerehabilitation can be preferred instead of face-to-face occupational therapy practices in accordance with the literature.

We gauged the level of occupational balance of the study subjects at the outset and at the end of the study by means of the OQB11 scale. Our results showed that a training program via telerehabilitation significantly improved occupational balance in refugee children; occupational balance is also important for the well-being of children. Being a refugee child can be seen as an epitomized example of a traumatized child, particularly if the child was exposed to violence and death in his or her native country. We now know well that past traumatic events might lead to psychological and cognitive sequelae. Several investigators have shown that these unfavorable experiences can result in emotional, cognitive, and behavioral alterations [31]. Due to their inability to organize themselves, children who have experienced war and have high stress levels suffer learning difficulties, attention deficiencies, academic failure, and occupational balance problems [5,32,33]. In this context, Copley et al.,’s recommendations [33] demonstrated the need for the intervention program that we developed. Several studies demonstrated that occupational imbalance had a negative impact on school performance [34]. To the best of our knowledge, no study to date has attempted to examine the effects of an online activity-based training on the occupational balance of refugee children. We think that in the content of our intervention program, routinely regulating individual programs and group physical activities, cognitive application games and group games that facilitate competition support children’s capacities of “doing”, “being”, and “becoming” in the philosophy of occupational therapy. The synthesis of doing, being, and becoming is critical to an individual’s health and well-being, which can be achieved through meaningful activities [35]. With the increase in improving this synthesis, the improvement in occupational performance may have increased the well-being in children.

The results of our research show that the well-being of refugee children who are included in an occupational therapy program through telerehabilitation has increased. Positive feelings and thoughts, the absence of negative emotions, life satisfaction, fulfillment, and positive functioning are all components of well-being, which has been linked to a variety of health, work, family, and financial benefits [36]. Thus, refugee children generally have less than optimal general well-being [37]. Education and schools have a significant role in the well-being of vulnerable children. However, the COVID-19 lockdown prevented school attendance in person and this might potentially have impaired the well-being of the refugee children. Park and colleagues found that occupational balance was an independent associate of subjective health, quality of life, and health-related variables [38]. In fact, some studies have demonstrated that occupational therapy and increased occupational performance were associated with increased well-being and quality of life in various populations [39]. In the literature, it has been shown that mental health practices applied to refugee children cause improvement in the psychological well-being of children. For instance, forty-seven refugee children were included in the study by creating a control group in the school-based mental health intervention and at the end of the study it was observed that the children’s psychological functioning improved, especially in the emotional field and peer relations [40]. In another study by Stein et al., mental health intervention was applied to school-age refugee children and a decrease in post-traumatic stress disorder and depression symptoms was observed in children after the intervention [41]. As seen above, various intervention programs have been applied to refugee children, but no occupational therapy program has been found. The occupational therapy program we implemented included a timetable that covered the whole day of the children from morning to evening. Thanks to this activity-containing timeline, the children learned how to structure their day. At the same time, practices that ensure a balanced distribution in different activity areas throughout the day allowed children to engage in activities in a controlled and regular manner in cognitive, psychosocial, and physical activity areas. For this reason, we think that the well-being of children included in the occupational therapy group will increase through telerehabilitation.

We observed that intrinsic motivation improved in refugee children who were included in the occupational therapy program through telerehabilitation. Interest in intrinsic motivation in the literature has been steadily increasing in the last decade. Intrinsic motivation is considered as an energized behavior emanating from an individual owing to an ingrained interest in the activity at hand. The importance of curricular strategies to enhance the intrinsic motivation of the children is emphasized [42]. A study of refugee children’s support networks was conducted in Kenya’s Dadaab refugee camp. Academic motivation was found to be influenced by non-material assistance such as encouragement and advocacy received through emotional and academic support from family and community members, teachers, and peers. This research demonstrates that even in circumstances of long-term displacement, support can raise motivation and that increased motivation can improve academic achievement through education or rehabilitation [43]. Several methods have been shown to increase intrinsic motivation of diverse people groups via occupational therapy applied within the scope of rehabilitation [44]. In addition to the difficulties that come with being a refugee child, we consider that intrinsic motivation is of crucial importance to keep the interests of the children high to facilitate learning, particularly during the boredom with family home life in COVID-19 lockdown. When we used occupational therapy via telerehabilitation in our study, the intrinsic motivation level of the children significantly increased in the intervention.

We found an increase in the quality of life of refugee children who participated in an occupational therapy program that included both individual and group activities. A person’s impressions of several life dimensions such as health, relationships, learning, and participation, among others, are referred to as quality of life [15]. Dangmann et al., investigated the influence of past traumatic events and new stressors in the resettled country on health-related quality of life (HRQoL) [45]. The study recruited 160 Syrian youth recently resettled in Norway. The results of the study showed that higher levels of past traumatic events adversely affected HRQoL. Current stressors had a negative impact as well. It was shown that psychological treatment via mental health specialist services had beneficial effects on mental health, quality of life, and resettlement life functioning in adult refugees who had war-related traumas [46]. The concept of quality of life encompasses physical, psychological, and psychosocial elements; one of these aspects is activity participation. Children can build their competences, attain mental and physical health, understand their talents and abilities, and form enduring meaningful relationships by participating in the activities. Children learn the skills they need to be successful and autonomous in school and society through involvement. As a result, involvement is becoming more widely acknowledged as one of the key aims of pediatric rehabilitation and it is thought to benefit children’s health, development, and quality of life [47]. The fact that our occupational therapy program developed in light of this evidence incorporates physical activities, competitive cognitive games, and group activities with both individual and social interaction suggests that it can be helpful in enhancing refugee children’s quality of life despite the lockdown of COVID-19.

Some limitations of our study deserve mention. Our intervention period was relatively short and, though we showed significant improvements in occupational balance, well being, intrinsic motivation, and quality of life, we did not specifically evaluate the impact of these favorable changes on learning ability or school success. In addition, the activities we used in the intervention were relatively limited. Leisure is one of the activity areas, however we may not have been able to sufficiently diversify the leisure activities of refugee students as much as the opportunities in their home environment.

Despite the limitations mentioned above, this study also has strengths. The favorable outcomes of this study not only offer interesting options to therapists to reduce occupational dysfunctions of refugee children, but also show that a new intervention for refugee children, which is extremely rare, has been demonstrated to be promising and it is suggested that similar interventions should be used more frequently in future research.

## 5. Conclusions

This was the first study investigating the effects of a customized occupational therapy training course via telerehabilitation on well-being, occupational balance, quality of life, and intrinsic motivation in Syrian refugee children also affected unfavorably by COVID-19 lockdown. Using occupational therapy via telerehabilitation in refugee children seems to be effective for decreasing occupational balance problems and enhancing well-being, intrinsic motivation, and quality of life.

## Figures and Tables

**Table 1:** General characteristics of the intervention and control groups.

	Groups (*n* = 52)		
	Intervention Group (*n* = 26)	Control Group (*n* = 26)	*p*-Value
Age (years)	13.5 (13–15)	13.5 (13–15)	0.796 ^m^
Number of siblings	2.5 (1–5)	3 (1–7)	0.150 ^m^
Duration of resettlement in Turkey	8 (5–10)	8 (5–10)	0.694 ^m^
Number of siblings			
<3 siblings	13 (50%)	9 (34.6%)	0.262 ^X2^
≥3 siblings	13 (50%)	17 (65.4%)	
Duration of resettlement in Turkey			
<8 years	9 (34.6%)	8 (30.8%)	0.768 ^X2^
≥8 years	17 (65.4%)	18 (69.2%)	

^X2^ Pearson Chi-Square, ^m^ Mann–Whitney U test. 3 and 8, number of siblings and duration of resettlement, respectively, are median values of the variables.

**Table 2:** One week example of group activities performed via Zoom videoconference.

	Group Activities		
	16:15–16:30	16:30–17:15	17:15–17:30
Monday	Explaining what they understand from the Turkish reading book they read, interpreting idiom/proverb (every day one child will do)	Memory game (everyone will say an object in turn and the next person will add the object of their choice to this chain by saying the words said before them first; those who forget or say incorrectly will be eliminated and the last remaining child will be the champion)	Simultaneously performing the movements demonstrated by the researcher online (sports activities)
Tuesday	Explaining what they understand from the Turkish reading book they read, interpreting idiom/proverb (every day one child will do)	They will create shapes from pasta and legumes online and paint them at their pleasure or decorate them with other materials, and at the end of the time the child who made the most beautiful will be determined and announced.	Simultaneously performing the movements demonstrated by the researcher online (sports activities)
Wednesday	Explaining what they understand from the Turkish reading book they read, interpreting of idiom proverb (every day one child will do)	The categories game	Simultaneously performing the movements demonstrated by the researcher online (sports activities)
Thursday	Explaining what they understand from the Turkish reading book they read, interpreting of idiom proverb (every day one child will do)	BOM game (For example, they will not say multiples of 6 and numbers that contain 6, but instead say BOM. It will be progressed starting from number 1. Those who make mistakes will be eliminated and the last person standing will be declared champion)	Simultaneously performing the movements demonstrated by the researcher online (sports activities)
Friday	Explaining what they understand from the Turkish reading book they read, interpreting of idiom proverb (every day one child will do)	Cooking together a selected meal at the same time while online	Simultaneously performing the movements demonstrated by the researcher online (sports activities)

**Table 3:** The chart depicting one week of occupational balance program.

	08:30–09:30	After 09:30	15:00–15:20	16:15–17:30	17:30–19:00	19:00–21:00	21:00–22:00	22:00–22:45	22:45–00:30
Monday	Waking up	EBA remote education classes and breakfast/rest	Learning idioms and proverbs (one each day)	Group training	Rest and meal time	Taking the quiz (50 questions) and doing homework given by the teachers at school	Playing mind-boosting cognitive games with the MentalUp app	Reading book	Rest and sleep
Tuesday	Waking up	EBA distance education courses and breakfast/rest	Learning idioms and proverbs (one each day)	Group training	Rest and meal time	Taking the quiz (50 questions) and doing homework given by the teachers at school	Playing mind-boosting cognitive games with the MentalUp app	Reading book	Rest and sleep
Wednesday	Waking up	EBA distance education courses and breakfast/rest	Learning idioms and proverbs (one each day)	Group training	Rest and meal time	Taking the quiz (50 questions) and doing homework given by the teachers at school	Playing mind-boosting cognitive games with the MentalUp app	Reading book	Rest and sleep
Thursday	Waking up	EBA distance education courses and breakfast/rest	Learning idioms and proverbs (one each day)	Group training	Rest and meal time	Taking the quiz (50 questions) and doing homework given by the teachers at school	Playing mind-boosting cognitive games with the MentalUp app	Reading book	Rest and sleep
Friday	Waking up	EBA distance education courses and breakfast/rest	Learning idioms and proverbs (one each day)	Group training	Rest and meal time	Taking the quiz (50 questions) and doing homework given by the teachers at school	Playing mind-boosting cognitive games with the MentalUp app	Reading book	Rest and sleep
Saturday	Waking up	-	Learning idioms and proverbs (one each day)	-	Rest and meal time	Taking the quiz (50 questions) and doing homework given by the teachers at school	Playing mind-boosting cognitive games with the MentalUp app	Reading book	Rest and sleep
Sunday	-	-	-	-	-	-	-	-	-

**Table 4:** General characteristics of the intervention and control groups.

	Groups (*n* = 52)		
	Intervention Group (*n* = 26)	Control Group (*n* = 26)	*p*-Value
Age (years)	13.5 (13–15)	13.5 (13–15)	0.796 ^m^
Number of siblings	2.5 (1–5)	3 (1–7)	0.150 ^m^
Duration of resettlement in Turkey	8 (5–10)	8 (5–10)	0.694 ^m^
Number of siblings			
<3 siblings	13 (50%)	9 (34.6%)	0.262 ^X2^
≥3 siblings	13 (50%)	17 (65.4%)	
Duration of resettlement in Turkey			
<8 years	9 (34.6%)	8 (30.8%)	0.768 ^X2^
≥8 years	17 (65.4%)	18 (69.2%)	

^X2^ Pearson Chi-Square, ^m^ Mann–Whitney U test. 3 and 8, number of siblings and duration of resettlement, respectively, are median values of the variables.

**Table 5:** Comparison of the baseline scores of the study scales.

	Groups (*n* = 52)		
	Intervention Group (*n* = 26)	Control Group (*n* = 26)	*p*-Value
Occupational Balance Questionnaire (OBQ11)	17.5 ± 4.6	18.2 ± 4.7	0.634 ^†^
Well-Star scale (WSS)	73.9 ± 22.2	73.2 ± 21.6	0.910 ^†^
Intrinsic Motivation Scale (IMS)	79.7 ± 21.6	81.4 ± 18.5	0.763 ^†^
Pediatric Quality of Life Inventory (PedsQL)			
Total scale score (TSS)	61.4 ± 7.0	58.9 ± 8.5	0.253 ^†^
Physical health summary score (PHSS)	65.6 (50.0–84.4)	64.1 (50.0–82.8)	0.740 ^m^
Psychosocial health summary score (PsHSS)	57.8 ± 7.1	54.3 ± 9.7	1.150 ^†^
Emotional functioning score (EFS)	59.6 ± 11.3	57.5 ± 12.8	0.530 ^†^
Social functioning score (SFS)	57.7 ± 9.6	55.0 ± 11.5	0.364 ^†^
School functioning score (ScFS)	52.5 (40.0–75.0)	50.0 (20.0–75.0)	0.140 ^m^

^†^ independent samples t-test, ^m^ Mann–Whitney U test.

**Table 6:** Summarizes baseline and post-intervention scores of study scales for the control and intervention groups.

	Scores		
INTERVENTION GROUP			
	**Before the Study**	**After the Study**	***p*-Value**
Occupational Balance Questionnaire (OBQ)	17.5 ± 4.6	24.9 ± 5.0	<0.001 *
Well-Star scale (WSS)	73.9 ± 22.2	88.9 ± 17.1	<0.001 *
Intrinsic Motivation scale (IMS)	79.7 ± 21.6	95.6 ± 18.4	<0.001 *
Pediatric Quality of Life Inventory (PedsQL)			
Total scale score (TSS)	61.4 ± 7.0	74.0 ± 7.2	<0.001 *
Physical health summary score (PHSS)	65.6 (50.0–84.4)	78.1 (50–100)	<0.001 ^w^
Psychosocial health summary score (PsHSS)	57.8 ± 7.1	70.4 ± 8.2	<0.001 *
Emotional functioning score (EFS)	59.6 ± 11.3	69.4 ± 11.0	<0.001 *
Social functioning score (SFS)	57.7 ± 9.6	68.3 ± 7.6	<0.001 *
School functioning score (ScFS)	52.5 (40.0–75.0)	75.0 (50.0–100.0)	<0.001 ^w^
CONTROL GROUP			
	**Before the Study**	**After the Study**	***p*-Value**
Occupational Balance Questionnaire (OBQ)	18.2 ± 4.7	18.4 ± 4.8	0.740 *
Well-Star scale (WSS)	73.2 ± 21.6	72.2 ± 19.2	0.421 *
Intrinsic Motivation scale (IMS)	81.4 ± 18.5	79.2 ± 19.2	0.093 *
Pediatric Quality of Life Inventory (PedsQL)			
Total scale score (TSS)	58.9 ± 8.5	57.2 ± 7.7	0.116 *
Physical health summary score (PHSS)	64.1 (50.0–82.8)	63.6 (50–8)	0.702 ^w^
Psychosocial health summary score (PsHSS)	54.3 ± 9.7	51.2 ± 8.2	0.004 *
Emotional functioning score (EFS)	57.5 ± 12.8	56.0 ± 10.2	0.212
Social functioning score (SFS)	55.0 ± 11.5	53.7 ± 11.3	0.244
School functioning score (ScFS)	50 (20–75)	45 (25–75)	0.011 ^w^

* paired sample t-test, ^w^ Wilcoxon signed-rank test.

**Table 7:** Comparison of “score differences” between the intervention and control groups. Score differences were obtained as subtracting the baseline score from the post-intervention score for individual scales.

	Groups (*n* = 52)		
	Intervention Group (*n* = 26)	Control Group (*n* = 26)	*p*-Value
Occupational Balance Questionnaire (OBQ)	7.38 ± 3.31	0.19 ± 2.93	<0.001 ^†^
Well-Star scale (WSS)	13.5 (−2–43)	1 (−15–8)	<0.001 ^m^
Intrinsic Motivation scale (IMS)	17 (−4–34)	−2.5 (−16–6)	<0.001 ^m^
Pediatric Quality of Life Inventory (PedsQL)			
Total scale score (TSS)	12.67 ± 5.66	−1.69 ± 5.19	<0.001 ^†^
Physical health summary score (PHSS)	13.28 ± 9.53	−0.24 ± 8.38	<0.001 ^†^
Psychosocial health summary score (PsHSS)	13.33 (6.67–23.33)	−2.50 (−16.67–5)	<0.001 ^m^
Emotional functioning score (EFS)	10 (0–20)	−2.50 (−20–5)	<0.001 ^m^
Social functioning score (SFS)	10 (0–25)	0 (−15–5)	<0.001 ^m^
School functioning score (ScFS)	15 (0–50)	0 (−25–5)	<0.001 ^m^

^†^ independent samples t-test, ^m^ Mann–Whitney U test.

## Data Availability

The dataset analyzed in this study is available from the University of Health Sciences Turkey (sumeyye.belhancelik@sbu.edu.tr) on reasonable request.

## References

[1] UNICEF Press Release 11 March 2021. https://www.unicef.org/press-releases/syria-conflict-10-years-90-cent-children-need-support-violence-economic-crisis-and.

[2] Data from DGMM. https://en.sg.gov.tr/irregular-migration-statistics.

[3] UNICEF Turkey Humanitarian Situation Report No. 40, Reporting Period: January—December 2020. UNICEF Turkey. https://www.unicef.org/turkey/en.

[4] Sahin E., Dagli T.E., Acarturk C., Dagli F.S. (2020). Vulnerabilities of Syrian refugee children in Turkey and actions taken for prevention and management in terms of health and wellbeing. Child Abuse Negl..

[5] Kaplan I., Stolk Y., Valibhoy M., Tucker A., Baker J. (2016). Cognitive assessment of refugee children: Effects of trauma and new language acquisition. Transcult. Psychiatry.

[6] De Haene L., Grietens H., Verschueren K. (2007). From symptom to context: A review of the literature on refugee children’s mental health. Hell. J. Psychol..

[7] Görmez V., Kılıç H.N., Örengül A.C., Demir M.N., Mert E.B., Makhlouta B., Kınık K., Semerci B. (2017). Evaluation of a school-based, teacher-delivered psychological intervention group program for trauma-affected Syrian refugee children in Istanbul, Turkey. Psychiatry Clin. Psychopharmacol..

[8] Rodriguez-Fernandez P., Gonzalez-Santos J., Santamaria-Pelaez M., Soto-Cámara R., González-Bernal J. (2021). Exploring the Occupational Balance of Young Adults during Social Distancing Measures in the COVID-19 Pandemic. Int. J. Environ. Res. Public Health.

[9] Morville A.-L., Erlandsson L.-K. (2013). The experience of occupational deprivation in an asylum centre: The narratives of three men. J. Occup. Sci..

[10] Wagman P., Håkansson C., Björklund A. (2012). Occupational balance as used in occupational therapy: A concept analysis. Scand. J. Occup. Ther..

[11] Teraoka M., Kyougoku M. (2015). Development of the final version of the classification and assessment of occupational dysfunction scale. PLoS ONE.

[12] Nasaba R., Tindyebwa D., Musiime V., Iriso R., Ingabire R., Nansera D., Duffy M. (2018). Handbook on Counselling and Psychosocial Care for Children and Adolescents Living with and Affected by HIV in Africa.

[13] Akoğlu G., Karaaslan B.T. (2020). COVID-19 ve izolasyon sürecinin çocuklar üzerindeki olası psikososyal etkileri. İzmir Katip Çelebi Üniversitesi Sağlık Bilimleri Fakültesi Derg..

[14] Aslan M., Doğan S. (2020). Dişsal motivasyon, içsel motivasyon ve performans etkileşimine kuramsal bir bakiş. Süleyman Demirel Üniversitesi Vizyoner Derg..

[15] The Whoqol Group (1998). The World Health Organization Quality of Life Assessment (WHOQOL): Development and general psychometric properties. Soc. Sci. Med..

[16] Nobari H., Fashi M., Eskandari A., Villafaina S., Murillo-Garcia Á., Pérez-Gómez J. (2021). Effect of COVID-19 on Health-Related Quality of Life in Adolescents and Children: A Systematic Review. Int. J. Environ. Res. Public Health.

[17] Testa M.A., Simonsson D.C. (1996). Assessment of quality-quality-of-life outcomes. N. Engl. J. Med..

[18] Wallander J.L., Koot H.M. (2016). Quality of life in children: A critical examination of concepts, approaches, issues, and future directions. Clin. Psychol. Rev..

[19] Pizzi M.A., Richards L.G. (2017). Promoting Health, Well-Being, and Quality of Life in Occupational Therapy: A Commitment to a Paradigm Shift for the Next 100 Years. Am. J. Occup. Ther..

[20] Gefen N., Steinhart S., Beeri M., Weiss P. (2021). Lessons Learned during a Naturalistic Study of Online Treatment for Pediatric Rehabilitation. Int. J. Environ. Res. Public Health.

[21] Schulz K.F., Altman D.G., Moher D. (2010). CONSORT 2010 statement: Updated guidelines for reporting parallel group randomized trials. Trials.

[22] Gunal A., Pekcetin S., Demirturk F., Şenol H., Håkansson C., Wagman P. (2020). Validity and reliability of the Turkish Occupational Balance Questionnaire (OBQ11-T). Scand J. Occup. Ther..

[23] Owen F., Doğan T.K., Çelik N.D., Owen D.W. (2016). Development of The Well Star Scale İyilik Hali Yıldızı Ölçeği’nin geliştirilmesi. J. Hum. Sci..

[24] Duman İ., Horzum M.B., Randler C. (2020). Adaptation of the intrinsic motivation inventory to Turkish. Int. J. Psychol. Educ. Stud..

[25] Memik N.C., Agaoglu B., Coskun A., Uneri O.S., Karakaya I. (2007). The validity and reliability of the Turkish Pediatric Quality of Life Inventory for children 13–18 years old. Turk. Psikiyatr. Derg..

[26] Varni J.W., Seid M., Kurtin P.S. (2001). PedsQL 4.0: Reliability and validity of the Pediatric Quality of Life Inventory version 4.0 generic core scales in healthy and patient populations. Med. Care.

[27] Doumit R., Kazandjian C., Militello L.K. (2020). COPE for Adolescent Syrian Refugees in Lebanon: A Brief Cognitive-Behavioral Skill-Building Intervention to Improve Quality of Life and Promote Positive Mental Health. Clin. Nurs. Res..

[28] Gustafsson L. (2020). Occupational therapy has gone online: What will remain beyond COVID-19?. Aust. Occup. Ther. J..

[29] Ganesan B., Fong K.N.K., Meena S.K., Prasad P., Tong R.K.Y. (2021). Impact of COVID-19 pandemic lockdown on occupational therapy practice and use of telerehabilitation—A cross sectional study. Eur. Rev. Med. Pharmacol. Sci..

[30] Dahl-Popolizio S., Carpenter H., Coronado M., Popolizio N.J., Swanson C. (2020). Telehealth for the provision of occupational therapy: Reflections on experiences during the COVID-19 pandemic. Int. J. Telerehabil..

[31] Pechtel P., Pizzagalli D.A. (2011). Effects of early life stress on cognitive and affective function: An integrated review of human literature. Psychopharmacology.

[32] Al-Krenawi A., Graham J.R. (2012). The impact of political violence on psychosocial functioning of individuals and families: The case of Palestinian adolescents. Child Adolesc. Ment. Health.

[33] Copley J., Turpin M., Gordon S., McLaren C. (2011). Development and evaluation of an occupational therapy program for refugee high school students. Aust. Occup. Ther. J..

[34] Raveicaa G., Raveicab I.C., Ciucurel M.M. (2012). Occupational Balance in Children of 8–10 Years and its Influence on School Performance. Proced. Soc. Behav. Sci..

[35] Hitch D., Pepin G. (2021). Doing, being, becoming and belonging at the heart of occupational therapy: An analysis of theoretical ways of knowing. Scand. J. Occup. Ther..

[36] Lyubomirsky S., King L., Diener E. (2005). The benefits of frequent positive affect: Does happiness lead to success?. Psychol. Bull..

[37] Hamdan-Mansour A.M., Abdel Razeq N.M., AbdulHaq B., Arabiat D., Khalil A.A. (2017). Displaced Syrian children’s reported physical and mental wellbeing. Child Adolesc. Ment. Health.

[38] Park S., Lee H.J., Jeon B.J., Yoo E.-Y., Kim J.-B., Park J.-H. (2021). Effects of occupational balance on subjective health, quality of life, and health-related variables in community-dwelling older adults: A structural equation modeling approach. PLoS ONE.

[39] Yang S.Y., Wang J.D., Chang J.H. (2020). Occupational therapy to improve quality of life for colorectal cancer survivors: A randomized clinical trial. Supportive Care Cancer.

[40] Fazel M., Doll H., Stein A. (2009). A school-based mental health intervention for refugee children: An exploratory study. Clin. Child Psychol. Psychiatry.

[41] Stein B.D., Jaycox L.H., Kataoka S.H., Wong M., Tu W., Elliott M.N., Fink A. (2003). A mental health intervention for schoolchildren exposed to violence: A randomized controlled trial. JAMA.

[42] Chaudhuri J.D. (2020). Stimulating Intrinsic Motivation in Millennial Students: A New Generation, a New Approach. Anat. Sci. Educ..

[43] Dryden-Peterson S. (2016). Refugee education in countries of first asylum: Breaking open the black box of pre-resettlement experiences. Theory Res. Educ..

[44] Wu C.Y. (2001). Facilitating intrinsic motivation in individuals with psychiatric illness: A study on the effectiveness of an occupational therapy intervention. Occup. Ther. J. Res..

[45] Dangmann C., Solberg O., Andersen P.N. (2021). Health-related quality of life in refugee youth and the mediating role of mental distress and post-migration stressors. Qual. Life Res..

[46] Opaas M., Wentzel-Larsen T., Varvin S. (2020). The 10-year course of mental health, quality of life, and exile life functioning in traumatized refugees from treatment start. PLoS ONE.

[47] Shikako-Thomas K., Dahan-Oliel N., Shevell M., Law M., Birnbaum R., Rosenbaum P., Poulin C., Majnemer A. (2012). Play and be happy? Leisure participation and quality of life in school-aged children with cerebral palsy. Int. J. Pediatrics.

